# Long-Term Eosinophil Depletion: A Real-World Perspective on the Safety and Durability of Benralizumab Treatment in Severe Eosinophilic Asthma

**DOI:** 10.3390/jcm14010191

**Published:** 2024-12-31

**Authors:** Francesco Menzella, Mariarita Marchi, Marco Caminati, Micaela Romagnoli, Claudio Micheletto, Matteo Bonato, Giuseppe Idotta, Manuele Nizzetto, Giuseppina D’Alba, Massimiliano Cavenaghi, Michela Bortoli, Bianca Beghè, Laura Pini, Roberto Benoni, Gianluca Casoni, Rodolfo Muzzolon, Lucio Michieletto, Annamaria Bosi, Andrea Mastrototaro, Adela Diamandi, Mara Nalin, Gianenrico Senna

**Affiliations:** 1 Pulmonology Unit, S. Valentino Hospital, Montebelluna (TV), AULSS2 Marca Trevigiana, 31100 Treviso, Italy; annamaria.bosi@aulss2.veneto.it; 2 Respiratory Unit, Cittadella Hospital, AULSS6 Euganea, 35138 Padua, Italy; mariarita.marchi@aulss6.veneto.it (M.M.); michela.bortoli@aulss6.veneto.it (M.B.); 3 UOC Allergologia-Asma Center, University of Verona, 37129 Verona, Italy; marco.caminati@univr.it (M.C.); andrea.mastrototaro@univr.it (A.M.); gianenrico.senna@univr.it (G.S.); 4 Pulmonology Unit, Cà Foncello Hospital, AULSS2 Marca Trevigiana, 31100 Treviso, Italy; micaela.romagnoli@aulss2.veneto.it (M.R.); matteo.bonato@aulss2.veneto.it (M.B.); 5 Pulmonology Unit, Verona Integrated University Hospital, 37134 Verona, Italy; claudio.micheletto@aovr.veneto.it; 6 Pulmonology Unit, San Bortolo Hospital, AULSS6, 36100 Vicenza, Italy; giuseppe.idotta@aulss8.veneto.it (G.I.); massimiliano.cavenaghi@aulss8.veneto.it (M.C.); 7 Pulmonology Unit, Dolo-Mirano Hospital, AULSS3 Serenissima, 30122 Venice, Italy; manuele.nizzetto@aulss3.veneto.it (M.N.); giuseppina.dalba@aulss3.veneto.it (G.D.); 8 Department of Respiratory Diseases, University of Modena and Reggio Emilia, 41121 Modena, Italy; bianca.beghe@unimore.it; 9 Department of Emergencies and High Specialties, Azienda Socio Sanitaria Territoriale (ASST) Spedali Civili di Brescia, 25123 Brescia, Italy; laura.pini@unibs.it; 10 Public Health and Infectious Diseases Department, Sapienza University of Rome, 00185 Rome, Italy; roberto.benoni@iss.it; 11 National Center for Global Health, Italian National Institute of Health (Istituto Superiore di Sanità), 00161 Rome, Italy; 12 Pneumology Unit, Hospital of Rovigo, 45100 Rovigo, Italy; gianluca.casoni@aulss5.veneto.it (G.C.); mara.nalin@aulss5.veneto.it (M.N.); 13 Pulmonology Unit, S. Martino Hospital, AULSS1 Dolomiti, 32100 Belluno, Italy; rodolfo.muzzolon@aulss1.veneto.it; 14 Respiratory Disease Unit, Department of Cardiac Toracic and Vascular Sciences, Ospedale dell’Angelo, AULSS3 Serenissima, 30122 Venice, Italy; lucio.michieletto@aulss3.veneto.it (L.M.); adela.diamandi@aulss3.veneto.it (A.D.)

**Keywords:** severe eosinophilic asthma, biological therapy, benralizumab, eosinophil depletion, long-term benralizumab safety, long-term benralizumab effectiveness, real-world study

## Abstract

**Background/Objectives:** Benralizumab is an anti-IL-5 receptor alpha monoclonal antibody that induces the near-complete depletion of eosinophils. This study aimed to evaluate the long-term safety and effectiveness of benralizumab in patients with severe eosinophilic asthma (SEA) over an extended 48-month follow-up period, offering one of the longest real-world perspectives available. **Methods:** This was a single-arm, retrospective, observational, multicenter study involving 123 SEA patients treated with benralizumab at a dosage of 30 mg every 4 weeks for the first 3 doses and then every 8 weeks. The safety endpoints focused on the frequency and nature of adverse events and the likelihood that they were induced by benralizumab. The efficacy endpoints focused on lung function, asthma exacerbations and control, and oral corticosteroid use. **Results:** Benralizumab, consistent with its mechanism of action, led to the rapid and nearly complete depletion of eosinophils. In total, 26 adverse events (21.1%) were observed, with 1.6% related to the treatment and 0.8% categorized as serious (vagal hypotension). Bronchitis was the most common unrelated adverse event (15.4%), occurring between months 36 and 38. Importantly, benralizumab effectiveness and safety were maintained consistently across the 48-month duration, resulting in significant improvements in lung function and reductions in oral corticosteroid use and exacerbation frequency. **Conclusions:** Benralizumab demonstrated a favorable safety profile, comparable to previously published studies, with perdurable effectiveness in controlling SEA and reducing oral corticosteroid use. Finally, this study provides evidence that near-complete eosinophil depletion does not increase long-term safety risks and supports benralizumab as a reliable treatment option for SEA patients.

## 1. Introduction

Severe eosinophilic asthma (SEA) is characterized by extensive eosinophilic inflammation, triggered by the signaling of interleukin (IL)-5 through the IL-5 receptor alpha (IL-5Rα) [1,2,3,4,5,6]. Since increasing blood eosinophil (Eos) counts correlate with asthma severity, leading to a higher risk of exacerbations, poor asthma control, and declining lung function [7,8], the depletion of Eos is the primary therapeutic goal [9,10]. Two anti-IL-5 monoclonal antibodies (mepolizumab and reslizumab) and an anti-IL-5 receptor antibody (benralizumab) have been approved as add-on maintenance therapy for SEA [11]. Mepolizumab reduces Eos by approximately 83–86% [12], while benralizumab causes direct, rapid, nearly complete depletion of Eos from the peripheral blood and lungs [13]. Pharmacovigilance data on the clinical use of biological therapies in asthma confirm the safety of these treatments. Adverse events (AEs) are primarily injection-site reactions, nasopharyngitis, headaches, and hypersensitivity, while the association with malignancies, effects on the cardiovascular system, alopecia, and autoimmune conditions have not been verified and require further evaluation [1]. 

Benralizumab has demonstrated efficacy and safety in SEA patients, as shown in several clinical trials, real-world studies, and pharmacovigilance data [14,15,16]. The SIROCCO and CALIMA clinical trials showed the safety and efficacy of benralizumab versus placebo [14,15], while the ZONDA and PONENTE clinical trials and the PROMISE and ANANKE real-life studies also demonstrated the effectiveness of benralizumab in reducing the need for oral corticosteroids (OCS) [17,18,19,20]. The XALOC-1 real-world study assessed benralizumab’s effectiveness in 1002 adults with SEA during a 48-week follow-up, showing that the treatment reduced exacerbations and OCS use and improved symptom control and lung function [21]. Accordingly, the use of benralizumab has been recommended as an alternative noncorticosteroid treatment for acute exacerbations of SEA [22]. The ANDHI clinical trial demonstrated that benralizumab significantly improved the health-related quality of life, reduced the rate of exacerbations, enhanced lung function, and alleviated symptoms associated with nasal polyposis [23] Compared with mepolizumab, benralizumab resulted in greater improvements in lung function and exacerbation frequency [24], while also showing a faster onset of action [25].

Regarding the safety of benralizumab, the frequency of AEs during SIROCCO or CALIMA was comparable to that observed in the placebo arm, with no predisposition to other opportunistic infections [15,26] or malignancies [9]. The results from BORA and MELTEMI extension studies confirmed the sustained efficacy of benralizumab and its long-term safety. The MELTEMI study identified no new safety concerns for up to 5 years. The most common AEs in all groups were upper respiratory tract infections, headaches, and bronchitis [27,28].

To gain further insights into the long-term safety and effectiveness of benralizumab, this study aimed to provide real-world evidence collected over a comprehensive 48-month follow-up period, one of the longest durations reported in the literature for SEA. For this purpose, the ability of benralizumab to deplete Eos, the incidence of AEs, and the impact on lung function and asthma control were evaluated.

## 2. Patients and Methods

### 2.1. Ethical Considerations

The study protocol, identified as protocol number 1321/CE, received approval from the Ethics Committee Marca on 4 May 2023. The research adhered to the principles outlined in the Declaration of Helsinki and complied with all relevant local laws and regulations regarding the protection of personal data, including the General Data Protection Regulation (GDPR) (EU) 2016/679. Prior to participation, informed consent was obtained from all study participants.

### 2.2. Study Design and Study Intervention

This is a real-world, single-arm, retrospective observational multicenter study (Montebelluna, Cittadella, Treviso, Dolo, Verona, Vicenza, Rovigo, Mestre, Belluno, Modena, Brescia) to evaluate the safety and effectiveness of benralizumab 30 mg every 4 weeks for the first 3 doses, followed by every 8 weeks thereafter.

### 2.3. Patients

The diagnosis of severe, uncontrolled asthma in patients was conducted in accordance with the guidelines provided by the European Respiratory Society (ERS) and the American Thoracic Society (ATS). These patients were already receiving treatment involving medium- to high-dose inhaled corticosteroids (ICS) combined with long-acting β2-agonist (LABA) bronchodilators [29]. 

Benralizumab therapy was prescribed to adult patients following standard Italian clinical practice and the eligibility and reimbursement criteria established by the Agenzia Italiana del Farmaco (AIFA). Eligibility for benralizumab required patients to present with a blood count of eosinophils (Eos) ≥ 300 cells/μL, measured at any point before starting therapy, without concurrent oral corticosteroid (OCS) use. Beyond this requirement, patients needed to fulfill at least one of the following criteria: (1) a minimum of two asthma exacerbations within the previous 12 months despite receiving maximal-dose inhaled therapy, which necessitated systemic steroid use or hospitalization; or (2) continuous OCS therapy over the preceding year alongside maximal inhaled treatment [11]. Patients were recruited from the centers of Montebelluna, Treviso, Cittadella, Dolo, Belluno, Mestre, Vicenza, Verona, Rovigo, Brescia, and Modena between January 2019 and March 2024, and they were administered benralizumab subcutaneously at a dose of 30 mg, with the initial 3 doses given every 4 weeks, followed by subsequent doses every 8 weeks.

### 2.4. Data Collection

Demographic and disease data were collected from medical records based on assessments conducted during physicians’ routine clinical practice. Demographic variables included age, sex, body mass index (BMI), current and past smoking status, the age at diagnosis, and when the therapy started. Disease information comprised the presence of allergic rhinitis, chronic rhinosinusitis with nasal polyps (CRSwNP), bronchiectasis, atopy, hypersensitivity to acetylsalicylic acid (ASA) and nonsteroidal anti-inflammatory drug (NSAID), gastroesophageal reflux disease (GERD), and eosinophilic granulomatosis with polyangiitis (EGPA). Data on the severity of asthma included Eos count, asthma control test (ACT) score, pre-bronchodilator forced expiratory volume in 1 s (pre-BD FEV_1_), pre-bronchodilator forced vital capacity (pre-BD FVC), pre-bronchodilator forced expiratory flow (pre-BD FEF), fractional exhaled nitric oxide (FeNO), which is a non-invasive biomarker for allergic and/or eosinophilic airway inflammation in patients with asthma, and the dosage of OCS. Additionally, data on cardiovascular, metabolic, and/or neuropsychiatric comorbidities were collected.

### 2.5. Analysis of Safety and Efficacy Endpoints

The long-term safety and tolerability endpoints included the occurrence of AEs, serious AEs (SAEs), hypersensitivity, and immunogenicity. SAEs are those events that are life-threatening, require inpatient hospitalization or a prolongation of existing hospitalization, or cause death, disability, or incapacity. The number and percentage of AEs and the likelihood that they were caused by benralizumab therapy were determined.

Long-term effectiveness endpoints included the depletion of blood Eos and the consequent improvement in lung function, analyzed through FEV_1_ and FVC. Effectiveness was also evaluated by analyzing the level of asthma control using the Asthma Control Questionnaire 6 (ACQ-6) and ACT. Additionally, the frequency of relapses and hospitalizations, the daily dosage of OCS (expressed as prednisone-equivalent mg), and the proportion of patients discontinuing OCS usage were analyzed.

### 2.6. Statistical Analysis

A descriptive statistical analysis was conducted to examine patients’ baseline characteristics. Categorical variables were summarized using percentages and frequency rates, while continuous variables were presented as medians with interquartile ranges. Differences in the distribution of safety outcomes across various follow-up time points were evaluated using the chi-squared test or Fisher’s exact test, as appropriate. Respiratory function parameters were analyzed using a linear mixed-effects model for longitudinal data, incorporating subject-specific random effects to estimate changes in target outcomes over the follow-up period. While this sample size reflects the challenges of recruiting patients with SEA, it should be considered when interpreting the results.

Statistical analyses were performed using R v4.3. *p* < 0.05 was considered significant.

## 3. Results

### 3.1. Characteristics of the Population Enrolled in the Study

A total of 123 patients participated in the study, of whom 69 (56.1%) were female. The median age at diagnosis was 43.5 years, while the median age at the initiation of benralizumab was 61 years. The median BMI was 26.0, and 67.5% of patients did not smoke. The pre-BD FEV_1_ and FeNO values were 71.61 and 59.72, respectively; the ACT score was 15.04, and the Eos count was 646.54 cells/mm^3^. Overall, 33.3% of patients had allergic rhinitis, 56.9% had CRSwNP, 36.6% had GERD, and 5.7% had EGPA; bronchiectasis was present in 26.0%, atopy in 41.5%, and hypersensitivity to ASA/NSAIDs in 12.2%. Additionally, 40.7%, 27.6%, and 8.1% had cardiovascular, metabolic, or neuropsychiatric comorbidities, respectively (Table 1).

### 3.2. Safety Outcomes of Depleting Eosinophils with Benralizumab

**Figure 1:**
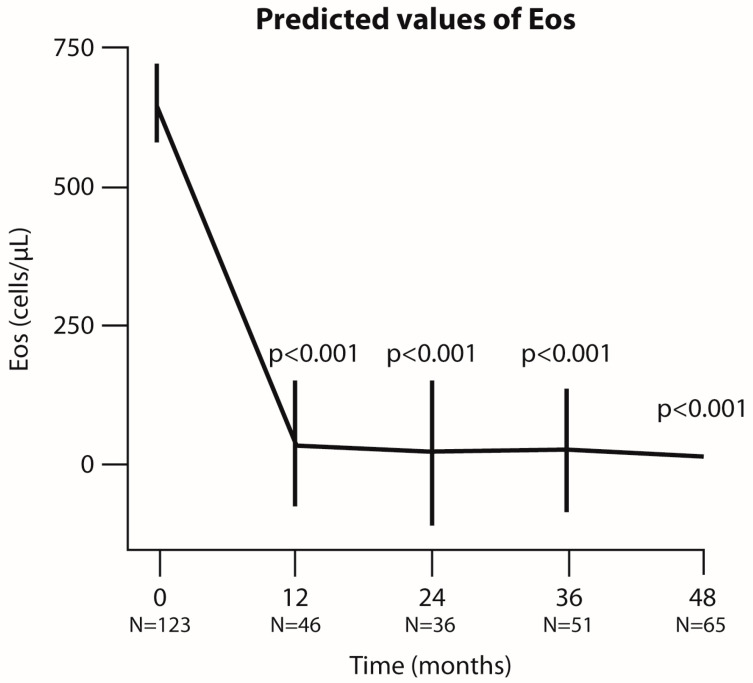
**Analysis of eosinophil count at each time point of the follow-up period (in months).** Graphical representation of eosinophil (Eos) values predicted by the linear mixed-effects (LMER) model. The bars indicate the 95% confidence interval (CI), and N represents the number of patients analyzed at each time point. *p*-values refer to the comparison between each time point and baseline.

Benralizumab significantly decreased the eosinophils (Eos) count, confirming its effectiveness in inhibiting IL-5Rα and inducing Eos apoptosis. Eos depletion was nearly complete at 36 months (*p* < 0.001) and maintained through 48 months (*p* < 0.001, Figure 1).

During the 48-month follow-up period, adverse events (AEs) occurred in 26 patients (21.1%), with only two patients (1.6%) who developed AEs related to benralizumab treatment. The two drug-related reactions occurred at baseline or near administration time and included nausea (1, 0.8%) and urticaria (1, 0.8%). A reaction potentially related to the therapy was vagal hypotension (1, 0.8%); however, its association with benralizumab use was not confirmed. None of the patients who experienced these AEs discontinued benralizumab treatment. Most infections reported as unrelated AEs were bronchitis (n = 4/26, 15.4%) cases that developed between 36 and 38 months. No differences were found in the risk of developing AEs based on sex (*p* = 0.259), age (*p* = 0.192), BMI (*p* = 0.846), smoking status (*p* = 0.662), allergic rhinitis (*p* = 0.825), CRSwNP (*p* = 0.791), or bronchiectasis (*p* = 0.658; Table 2). Importantly, the 48-month safety profile showed no evidence of cumulative AEs.

### 3.3. Effectiveness Endpoints

**Figure 2:**
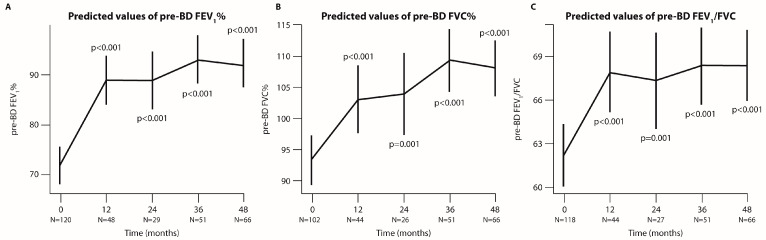
**Analysis of lung function measured through pre-BD FEV_1_, pre-BD FVC, and pre-BD FEV_1_/FVC at each time point of the follow-up period (in months).** Graphical representation of pre-BD FEV_1_ (**A**), pre-BD FVC% (**B**), and pre-BD FEV_1_/FVC (**C**) values predicted by linear mixed-effect (LMER) model. The bars indicate the 95% CI, and N represents the number of patients analyzed at each time point. *p*-values refer to the comparison between each time point and baseline.

In parallel with the highly efficient depletion of Eos, lung function improved considerably, as shown by the levels of pre-BD FEV_1_, pre-BD FVC, and pre-BD FEV_1_/FVC, which significantly increased at 36 months (*p* < 0.001) and were maintained at 48 months (*p* < 0.001, Figure 2).

**Figure 3:**
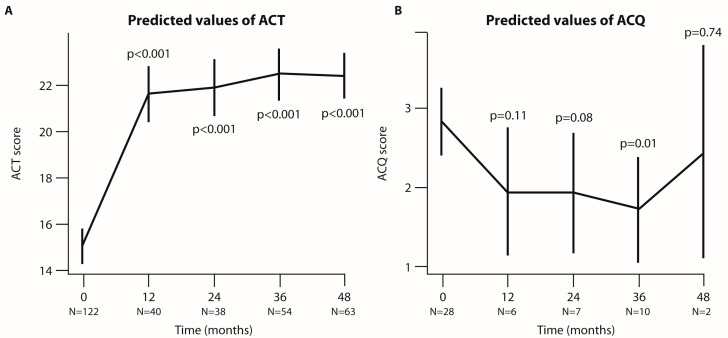
**Analysis of ACT and ACQ scores at each time point of the follow-up period (in months).** Graphical representation of ACT (**A**) and ACQ (**B**) predicted scores based on the linear mixed-effect (LMER) model. The bars indicate the 95% CI, and N represents the number of patients analyzed at each time point. *p*-values refer to the comparison between each time point and baseline.

ACT values increased significantly, and improvements were maintained at 36 and 48 months (*p* < 0.001, Figure 3A). ACQ scores were significantly lower at 36 months (*p* < 0.01; Figure 3B). These data show that Eos depletion by benralizumab achieves continuous improvement in asthma control and maintains a good safety profile in the long term.

**Figure 4:**
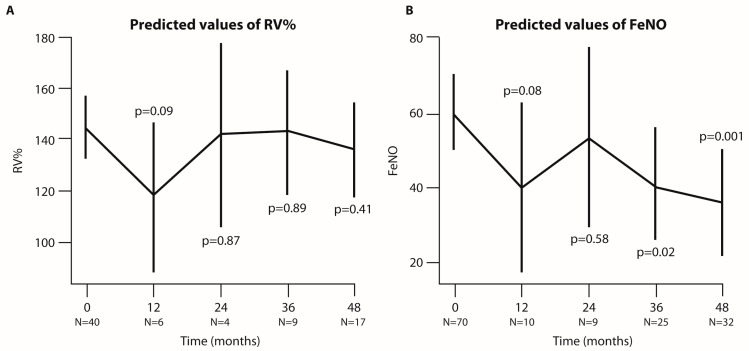
**Analysis of RV% and FeNO predicted values at each time point of the follow-up period (in months).** Graphical representation of RV% (**A**) and FeNO (**B**) predicted values based on the linear mixed-effect (LMER) model. The bars indicate 95% CI, and N represents the number of patients analyzed at each time point. *p*-values refer to the comparison between each time point and baseline.

No statistical differences were observed for RV% (Figure 4A), while FeNO decreased significantly (Figure 4B).

**Figure 5:**
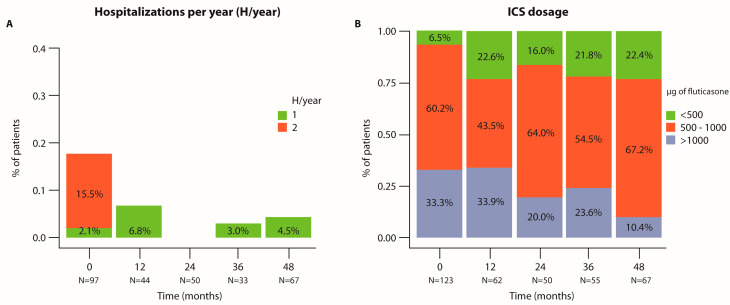
**Analysis of hospitalizations and dosage of ICS at each time point of the follow-up period (in months).** Graphical representation of the percentage of patients who were hospitalized 1 or 2 times during the follow-up period (**A**) and the percentage of patients who used during the follow-up period an ICS dosage (μg of fluticasone) <500, 500–1000, or >1000. (**B**). N represents the number of patients analyzed at each time point. The analysis was performed using the chi-square test.

Treatment with benralizumab reduced the number of hospitalizations, which occurred at baseline (before starting treatment) at a frequency of one hospitalization per year in 15.5% of patients and two hospitalizations per year in 2.1% of patients. At 48 months, these rates were 4.5% (one hospitalization) and 0.0% (two hospitalizations, Figure 5A). Additionally, the dosage of ICS decreased from over 1000 μg fluticasone in 33.3% of patients at baseline to 10.4% at 48 months, while the percentage of patients using a dosage <500 μg increased from 6.5% at baseline to 22.4% at 48 months (*p* = 0.007, Figure 5B).

**Figure 6:**
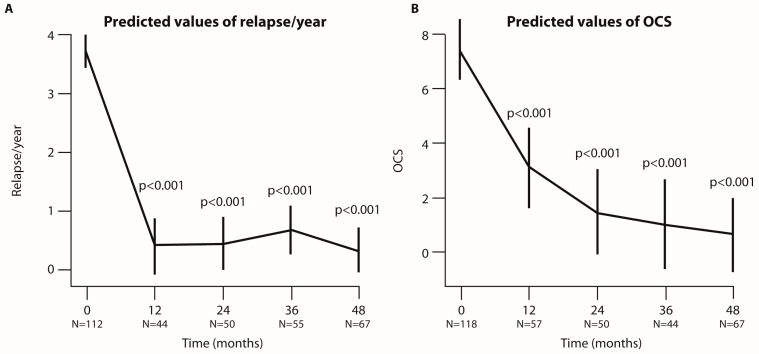
**Analysis of relapses per year and OCS dosage used at each time point of the follow-up period (in months).** Graphical representation of the number of relapses (**A**) and OCS dosage (**B**) predicted by linear mixed-effect (LMER) model. The bars indicate the 95% CI, and N represents the number of patients analyzed at each time point. *p*-values refer to the comparison between each time point and baseline.

The number of hospitalizations decreased significantly at 36 and 48 months (*p* < 0.001, Figure 6A). The dosage of OCS was reduced in 25/44 (56.8%) of patients, with a mean dosage reduction of 98% at 36 months (*p* < 0.001), and in 42/67 (62.7%) of patients, with a mean reduction of 96% at 48 months (*p* < 0.001, Figure 6B).

## 4. Discussion

SEA has a difficult-to-treat profile, and its control remains elusive despite the use of high-dose ICS combined with LABA bronchodilators [30]. Traditional therapy to prevent exacerbations consists of OCS, which, although effective, is associated with significant AEs [31]. Given the challenges in managing SEA, new biological agents have been developed over the past few decades to specifically target the eosinophilic inflammatory pathway. Biological drugs represent a major advancement in precision medicine for treating SEA, and their pharmacology is essential for selecting the most appropriate therapy for each patient, ensuring that the right drug is matched to the individual’s specific condition [32]. Therapies that deplete Eos have been approved for the treatment of SEA and include the anti-IL-5Rα monoclonal antibody benralizumab [11]. However, concerns have been raised about the safety of benralizumab therapy because of the role of peripheral tissue Eos in host defense against helminths [33,34], as well as viral, bacterial, and fungal pathogens [35,36,37,38] and their infiltration in several types of tumors [9,39]. The safety profile of benralizumab has been thoroughly evaluated in clinical trials and long-term studies, with no major concerns reported [28]. To gain further insights into the safety of benralizumab, this study collected and analyzed real-world data over a 48-month follow-up, one of the longest real-world studies reported to date. In this study population, benralizumab induced sustained Eos depletion, showing a good safety profile for up to 48 months. A total of 21.1% of patients reported AEs, while drug-related non-SAEs occurred in a small percentage (1.6%) and included nausea and urticaria. Only 0.8% of patients displayed an SAE, likely related to benralizumab treatment, which consisted of vagal hypotension. None of the AEs led to the discontinuation of treatment. No differences were found in AE development based on sex, age, BMI, smoking status, or comorbidities such as allergic rhinitis or bronchiectasis. Comparing our safety data with those of clinical trials, it emerged that, in this long-term real-world study, only 21.1% of patients experienced AEs, a significantly lower rate than the 62–75% observed in the clinical trials. However, these differences must be interpreted with caution, as inherent disparities between real-world and controlled trial settings may influence them. For instance, in comparison with the MELTEMI clinical trial, our study observed a lower incidence of AEs, particularly those associated with benralizumab. It is worth noting that this may reflect variations in study design, reporting practices, and patient populations rather than a definitive difference in safety profiles. In this work, the SAE incidence was also much lower (0.8% of patients reported vagal hypotension) than the frequency of 2–13.3% observed in the clinical trials. Bronchitis was more common in this study (15.4%) and was identified as an AE unrelated to benralizumab therapy (Table 3). Additionally, no cases of malignancies were observed over the 48-month period in this real-world study.

Comparison with the pharmacovigilance data and spontaneous reporting on benralizumab highlight some key findings. Across different datasets, the most frequently reported AEs were nasopharyngitis, headaches, and, as reported here, bronchitis. The rate of SAEs in pharmacovigilance databases is higher than that reported in this study, ranging from 11.5% to 18%, with rare but notable risks, such as anaphylaxis, vagal hypotension, and EGPA. Across all datasets and as observed in this study, infection risks were noted but not significantly increased. The frequency of malignancies was less than 1% in all datasets and 0% in this real-life study. Finally, discontinuation due to AEs was low in all datasets and 0% in this study (Table 4). Overall, the safety profile of benralizumab over the 48-month follow-up remains good, with consistent findings across spontaneous reports, clinical trials, and long-term follow-up studies. Notwithstanding, the smaller sample size and differences in study design should be considered when comparing results to larger clinical trials and pharmacovigilance datasets. These limitations warrant a cautious interpretation of the data.

Regarding the efficacy, by depleting Eos, benralizumab induced long-term disease control, improved lung function, and significantly reduced exacerbations, hospitalizations, and the ICS and OCS dosage required. The reduction in ICS dosage confirms the evidence from the SHAMAL study, although, in our study, the treatment period was significantly longer than in real-life settings [41]. Furthermore, a significant reduction in FeNO was observed, which could be due, as previously suggested [42], to the strong depletion of basophils and Eos induced by benralizumab. While benralizumab achieves near-complete Eos depletion compared to the partial depletion observed with mepolizumab, the literature indicates that both treatments have comparable safety profiles, with no increase in serious safety concerns [43]. Therefore, no increase in safety issues has been detected for benralizumab in comparison with mepolizumab to date. Moreover, the significant decrease in the need and dosage of OCS induced by benralizumab highlights the potential of this therapy to spare patients from AEs associated with corticosteroids.

We acknowledge that this study has several limitations, particularly, the small sample size. Since a total of 123 patients were included, there could be a reduction in the statistical power of the analysis that could limit the generalizability of the findings. Additionally, the retrospective design prevents direct comparisons with a placebo group or with other SEA treatments. Despite these limitations, the 48-month follow-up period provides valuable insights into the long-term safety and effectiveness of benralizumab, complementing data from clinical trials and pharmacovigilance databases.

To conclude, this 48-month real-world study confirms pharmacovigilance data and the results of clinical trials, showing a good safety profile of benralizumab, similar to that evidenced by other real-world data. Benralizumab-induced Eos depletion did not lead to an increase in AEs or SAEs compared with other biological therapies for asthma, such as mepolizumab. The rapid Eos depletion induced by benralizumab resulted in the highly effective management of SEA, allowing a swift reduction or discontinuation of OCS. Altogether, these findings suggest that benralizumab is a safe and effective treatment option for SEA, with evidence supporting its use over an extended duration.

## Figures and Tables

**Table 1:** Baseline characteristics of the whole sample.

Baseline Characteristics	Overall (N = 123), Median (IQR)/Mean (SD)/n (%)
Age at diagnosis (years)	43.5 (30.5–55.6)
Starting therapy age (years)	61.0 (54.0–70.0)
BMI (units)	26.0 (23.2–29.8)
Smoking status (N = 122)	
ex	36 (29.3%)
no	83 (67.5%)
yes	3 (2.4%)
Pre-BD FEV_1_ (% pred.)	71.61 (21.75)
Pre-BD FVC (% pred.)	93.41 (22.28)
Pre-BD FEV_1_/FVC (% pred.)	62.19 (12.60)
Pre-BD FEF% (% pred.)	42.72 (28.77)
ACT score	15.04 (4.68)
FeNO (ppb)	59.72 (52.52)
Eosinophils (cells/mm^3^)	646.54 (636.95)
Allergic rhinitis	41 (33.3%)
CRSwNP	70 (56.9%)
Bronchiectasis (n = 119)	32 (26.0%)
Atopy (n = 122)	51 (41.5%)
ASA NSAID hypersensitivity	15 (12.2%)
GERD	45 (36.6%)
EGPA (n = 122)	7 (5.7%)
Cardiovascular comorbidities	50 (40.7%)
Metabolic comorbidities	34 (27.6%)
Neuropsychiatric comorbidities (n = 122)	10 (8.1%)

ACT: asthma control test; ASA: acetylsalicylic acid; BMI: body mass index; CRSwNP: chronic rhinosinusitis with nasal polyps; EGPA: eosinophilic granulomatosis with polyangiitis; GERD: gastroesophageal reflux disease; NSAID: nonsteroidal anti-inflammatory drug; Pre-BD FEF: pre-bronchodilator forced expiratory flow; pre-BD FEV_1_: pre-bronchodilator forced expiratory volume in 1 s; pre-BD FVC: pre-bronchodilator forced vital capacity; FeNO: fractional exhaled nitric oxide.

**Table 2:** Adverse events reported during the 48-month follow-up following benralizumab treatment and stratified by follow-up time and causality assessment.

Adverse Events	Baseline, n (%)	Month 12, n (%)	Month 24, n (%)	Month 36, n (%)	Month 48, n (%)	Overall, n (%)
**Related**						
Nausea	1 (0.8%)	0 (0.0%)	0 (0.0%)	0 (0.0%)	0 (0.0%)	1 (0.8%)
Urticaria	1 (0.8%)	0 (0.0%)	0 (0.0%)	0 (0.0%)	0 (0.0%)	1 (0.8%)
**Likely**						
Vagal hypotension	1 (0.8%)	0 (0.0%)	0 (0.0%)	0 (0.0%)	0 (0.0%)	1 (0.8%)
**Not related**						
Altered coagulative diathesis	1 (0.8%)	0 (0.0%)	0 (0.0%)	0 (0.0%)	0 (0.0%)	1 (0.8%)
Bronchitis	0 (0.0%)	0 (0.0%)	0 (0.0%)	2 (1.6%)	2 (1.6%)	4 (3.3%)
Asthma exacerbation	0 (0.0%)	3 (2.4%)	4 (3.3%)	2 (1.6%)	5 (4.1%)	14 (11.4%)
Influenza	0 (0.0%)	0 (0.0%)	0 (0.0%)	1 (0.8%)	0 (0.0%)	1 (0.8%)
Polyarthritis	1 (0.8%)	0 (0.0%)	0 (0.0%)	0 (0.0%)	0 (0.0%)	1 (0.8%)
Pneumonia	0 (0.0%)	0 (0.0%)	0 (0.0%)	1 (0.8%)	1 (0.8%)	2 (1.6%)

**Table 3:** Comparison of the safety profile of benralizumab observed in this study with previous clinical trials in SEA patients.

Study	Design	Population	Duration	Key Safety Findings	Types of Adverse Events
SIROCCO [14]	Phase III, randomized, double-blind, placebo-controlled	1205 patients	48 weeks (12 months)	AEs: 71–73% in benralizumab vs. 78% in placebo SAEs: 12–13% (benralizumab) vs. 14% (placebo) Low discontinuation due to AEs (2%)	Common AEs: headaches (7–9%), nasopharyngitis (12%), upper respiratory tract infections (8–11%) Infusion-related reactions: rare (2–4%)
CALIMA [15]	Phase III, randomized, double-blind, placebo-controlled	1306 patients	56 weeks (13 months)	AEs: 74–75% in benralizumab vs. 78% in placebo Drug-related AE: 12–13% vs. 8% in placebo SAEs: 9–10% (benralizumab) vs. 14% (placebo) Similar rates of AEs between groups	Common AEs: nasopharyngitis (18–21%), headaches (8%), upper respiratory infections (7–8%) Infusion-related reactions: low incidence (2–3%)
ZONDA [17]	Phase III, randomized, double-blind, placebo-controlled	220 patients requiring chronic OCS	28 weeks (7 months)	AEs: 68–75% in benralizumab vs. 83% in placebo SAEs: 10% (benralizumab) vs. 19% (placebo) No increase in AEs during OCS reduction	Common AEs: nasopharyngitis (17%), worsening asthma (13%), and bronchitis (10%)
ANDHI [23]	Phase IIIb, open-label, observational	660 patients	24–32 weeks (6–8 months)	AEs: 62% in benralizumab SAEs: 8% in benralizumab Long-term safety was confirmed with no new signals	Common AEs: similar to SIROCCO and CALIMA Most frequent: infection-related AEs and headaches
MELTEMI [28]	Open-label extension study (2+ years of treatment)	1025 patients previously treated with benralizumab in prior trials	2 years	AEs: 64.6–84.6% in the benralizumab group vs. 45.9–87.7% in the placebo SAEs: 2.4–13.3% (benralizumab) vs. 4.5–14.2% in placebo Safety profile consistent, confirming long-term safety	Common AEs: nasopharyngitis (11.1–19.3%), headaches (5–12.6%), upper respiratory infections (1.6–8.9%), and bronchitis (3.6–9.2%) Infection-related AEs similar to previous studies
ANANKE [19]	Observational retrospective	162 patients	96 weeks	No new safety concerns reported	Not specifically reported
XALOC-1 [21]	Observational real-world study	1002 patients (380 biologic-experienced)	48 weeks (12 months)	No new safety concerns reported Not specifically detailed, but consistent with prior studies in safety profile	Not specifically reported
Long-term eosinophil depletion: a real-life perspective on safety and durability of benralizumab treatment in severe eosinophilic asthma	Long-term real-world study	123 patients previously treated with benralizumab	48 months (4 years)	AEs: 21.1% (26 total); only 1.6% related to treatment SAEs: 0.8% due to vagal hypotension, leading to discontinuation Bronchitis in 15.4% of infections, unrelated to treatment	Common AEs: bronchitis (15.4%), mostly between 36 and 38 months Related to infusion: nausea (0.8%) and urticaria (0.8%) SAE: vagal hypotension (0.8%), leading to discontinuation

AE: adverse event; OCS: oral corticosteroid; SAE: serious adverse event.

**Table 4:** Safety profile of benralizumab vs. pharmacovigilance databases.

Category	WHO Pharmacovigilance Database [40]	Spanish Pharmacovigilance Database [10]	Post-Marketing Surveillance and Spontaneous AE Reporting [9]	Long-Term Eosinophil Depletion: A Real-Life Perspective On Safety and Durability of Benralizumab Treatment in Severe Eosinophilic Asthma
Total cases reported	Over 5512 individual case safety reports (ICSRs)	588 reports in Spain	~36,680 patient-years (post-marketing exposure globally)	26 cases (21.1% of patients)
SAEs	SAEs in 29.5% and 1.3% of cases were life-threatening	18% of total cases categorized as serious	~11.5% (SIROCCO/CALIMA trials) and 16.9% in long-term studies (up to 2 years)	0.8%: vagal hypotension
Common AEs	General disorders (e.g., malaise, fatigue), injection-site reactions, nasopharyngitis, headaches, and hypersensitivity	Headaches (14.6%), pharyngitis (16.15%), fatigue (55 cases), pneumonia	Nasopharyngitis (16%), headaches (8.1%), bronchitis (7.9%)	Bronchitis (15.4%), nausea (0.8%), urticaria (0.8%)
Malignancies	Very low malignancy risk (<1%) noted in long-term data	Not emphasized	0.8% malignancy risk during 2-year trials (BORA)	No malignancies reported
Immune system reactions	Eosinophilic granulomatosis with polyangiitis (EGPA) risk noted	Anaphylaxis and hypersensitivity reactions noted	Hypersensitivity reactions (e.g., injection-site reactions) included in labeling	Vagal hypotension (0.8%)
Discontinuation rates	Not specifically reported	Not reported	~2% discontinuation due to AEs (SIROCCO/CALIMA trials), mostly mild reactions	None
Death reports	~3.2% related to serious adverse events	No specific death reports linked directly to benralizumab therapy	Deaths related to severe asthma complications in ~0.3% of long-term trial participants (BORA)	None

## Data Availability

The data underlying this study’s findings can be obtained from the corresponding author upon reasonable request.
